# Advanced Trauma Operative Management in Children

**DOI:** 10.3390/medicina59050963

**Published:** 2023-05-16

**Authors:** Federico Canavese

**Affiliations:** 1 Department of Pediatric Orthopedics, Lille University Center, Jeanne de Flandre Hospital, Avenue Eugène-Avinée, CEDEX, 59037 Lille, France; canavese_federico@yahoo.fr; Tel.: +33-3-20446867; Fax: +33-3-20444802; 2 Faculty of Medicine, Nord-de-France Lille University, 59000 Lille, France

Fractures in children and adolescents present a diagnostic and therapeutic challenge to the orthopedic surgeon as there are still many uncertainties in the scientific understanding of these injuries.

The causes of fractures in infants, children, and adolescents are heterogeneous in terms of etiology and epidemiology, as the anatomic regions of interest vary among different age groups. Despite the high incidence of pediatric fractures, there is still much debate about the optimal type of treatment of such injuries. Although both non-operative and surgical treatment techniques have developed tremendously in recent decades, current treatment strategies have limited scientific evidence. Particularly, traditional guidelines for the non-operative treatment of pediatric fractures have been challenged by surgical treatment, but it is unclear whether this translates into improved outcomes [1,2,3,4].

The current Special Issue on “Advanced Trauma Operative Management in Children” aims to improve knowledge about the epidemiology, treatment, and outcomes of pediatric fractures.

The epidemiology and distribution of pediatric fractures vary over time as they are influenced by multiple factors, such as climate, geography, and population characteristics. Monget et al. evaluated the distribution of pediatric fractures admitted to their institution in 1999 and 2019 and assessed the epidemiological differences 20 years later. They found that the types and locations of fractures have not changed over the past two decades, despite the increase in population and the change in trauma mechanism. In addition, the proportion of fractures managed non-operatively versus those treated surgically did not change between 1999 and 2019 [5].

Sapienza et al. have investigated the prone or supine position of the patient during the reduction and fixation of displaced supracondylar humerus fractures (SCHF) impact both the quality of reduction and the final outcome. The results of their systematic review highlighted that there was no statistically significant difference between the two positions with regard to the functional and radiographic outcome and the rate of complications. They concluded that the outcome of displaced SCHFs is generally excellent regardless of the position, prone or supine, in which the patient is positioned for surgery. In particular, they stressed that the choice of how to position the patient depends on the habit and experience of the surgeon and anesthesiologist performing the surgery [6].

Chronic Monteggia fractures (CMFs) can cause pain, functional disability, deformity, late ulnar nerve paralysis, and degenerative changes. The treatment of such injuries is challenging and yields unpredictable outcomes. In addition, the best surgical treatment method for this type of fracture has yet to be identified and remains a source of debate among specialists. Liu et al. compared the clinical and radiographic outcome of CMFs treated by ulnar osteotomy and monolateral external fixators with or without the angulation of the ulna during the distraction period. They found that a shorter time to achieve radial head reduction and fewer deformities of the ulna can be expected in patients undergoing the intraoperative restoration of ulnar alignment and gradual lengthening without angulation postoperatively. The authors concluded that ulna angulation might not be systematically needed in children with CMFs and gradual distraction could restore ulnar alignment [7].

The work of Wang et al. and Lu et al. focused on displaced femur neck fractures (DFNFs). DFNFs in children and adolescents are relatively rare and secondary to high-energy trauma. Due to the anatomy and blood supply of the proximal femur, such injuries are at risk of complications, despite timely and appropriate treatment. Wang et al. hypothesized that the number, size, and location of partially threaded cannulated screws might affect the fracture healing process of surgically treated DFNFs. However, they conducted a multicenter review of children and adolescents with DFNFs treated surgically and found that the healing process of such injuries is not affected by number, size, and location of hardware [8].

Lu et al. compared the outcome the clinical and radiographic outcomes of DFNFs in adolescents treated with a femoral neck system (28 patients) or a cannulated compression screw (30 patients). They reported that the femoral neck system has a lower complication rate and better functional outcome than cannulated compression screws and concluded that such a system is a valid alternative for treating DFNFs in adolescents [9].

Children are a distinct group of patients with very special and highly variable orthopedic problems. The presentation, management, and evolution of fractures in pediatric patients differ from those in the adult population, and special attention should be paid to them. With this in mind, the articles in the Special Issue “Advanced Trauma Operative Management in Children” are intended to contribute to the understanding of some of the pediatric trauma issues and offer valuable insights into the diagnosis and management of such conditions.
